# Regulation of Hippocampal and Behavioral Excitability by Cyclin-Dependent Kinase 5

**DOI:** 10.1371/journal.pone.0005808

**Published:** 2009-06-04

**Authors:** Ammar H. Hawasli, Della Koovakkattu, Kanehiro Hayashi, Anne E. Anderson, Craig M. Powell, Christopher M. Sinton, James A. Bibb, Donald C. Cooper

**Affiliations:** 1 Department of Psychiatry, University of Texas Southwestern Medical Center, Dallas, Texas, United States of America; 2 Department of Internal Medicine, University of Texas Southwestern Medical Center, Dallas, Texas, United States of America; 3 Department of Neurology, University of Texas Southwestern Medical Center, Dallas, Texas, United States of America; 4 Departments of Pediatrics, Neurology and Neuroscience, Baylor College of Medicine, Houston, Texas, United States of America; 5 Department of Psychology and Neuroscience, Institute for Behavioral Genetics, University of Colorado, Boulder, Colorado, United States of America; Mount Sinai School of Medicine, United States of America

## Abstract

Cyclin-dependent kinase 5 (Cdk5) is a proline-directed serine/threonine kinase that has been implicated in learning, synaptic plasticity, neurotransmission, and numerous neurological disorders. We previously showed that conditional loss of Cdk5 in adult mice enhanced hippocampal learning and plasticity via modulation of calpain-mediated *N*-methyl-D-aspartic acid receptor (NMDAR) degradation. In the present study, we characterize the enhanced synaptic plasticity and examine the effects of long-term Cdk5 loss on hippocampal excitability in adult mice. Field excitatory post-synaptic potentials (fEPSPs) from the Schaffer collateral CA1 subregion of the hippocampus (SC/CA1) reveal that loss of Cdk5 altered theta burst topography and enhanced post-tetanic potentiation. Since Cdk5 governs NMDAR NR2B subunit levels, we investigated the effects of long-term Cdk5 knockout on hippocampal neuronal excitability by measuring NMDAR-mediated fEPSP magnitudes and population-spike thresholds. Long-term loss of Cdk5 led to increased Mg^2+^-sensitive potentials and a lower threshold for epileptiform activity and seizures. Biochemical analyses were performed to better understand the role of Cdk5 in seizures. Induced-seizures in wild-type animals led to elevated amounts of p25, the Cdk5-activating cofactor. Long-term, but not acute, loss of Cdk5 led to decreased p25 levels, suggesting that Cdk5/p25 may be activated as a homeostatic mechanism to attenuate epileptiform activity. These findings indicate that Cdk5 regulates synaptic plasticity, controls neuronal and behavioral stimulus-induced excitability and may be a novel pharmacological target for cognitive and anticonvulsant therapies.

## Introduction

Cyclin-dependent kinase 5 (Cdk5), a proline-directed serine/threonine protein kinase, and its neuronal-specific activating cofactors have been implicated in numerous physiological and pathological processes in the mammalian nervous system [1]–[14]. Cdk5 has been implicated in hippocampal learning and synaptic plasticity [5], [7], [10], [15]–[17] and the pathogenesis of neurodegenerative disorders, such as Alzheimer's disease and neuropsychiatric illnesses, such as addiction [2], [15], [18], [19].

We previously reported that Cdk5 controls hippocampus-dependent learning and synaptic plasticity [7]. Conditional loss of Cdk5 improved performance in several hippocampal learning tasks and reduced the threshold for LTP induction. The enhancement in synaptic plasticity was due to increased NMDAR-mediated currents secondary to elevated surface expression of NR2B. Cdk5 was shown to facilitate the calpain-mediated degradation of NR2B upon activation of NMDARs. The regulation of NMDAR degradation appears to play a critical role in synaptic plasticity [20].

Our initial study revealed a number of positive effects that resulted from conditional loss of Cdk5 in the brains of adult mice [7], [15]. Inducible loss of Cdk5 in adult mice improved learning and increased NMDAR-mediated synaptic plasticity (2–4 weeks after knockout) [7], [15]. Here, we report the effects of adult, conditional Cdk5 loss on the induction of synaptic plasticity and hippocampal and stimulation-induced behavioral excitability. Since the loss of Cdk5 enhanced plasticity via increased NMDAR-mediated currents, we examined NMDAR-mediated field excitatory postsynaptic potential (fEPSP) magnitudes and population-spike thresholds in knockout (KO) and control (WT) mice. Although enhanced hippocampal plasticity is associated with increased learning we hypothesized that synaptic plasticity associated with loss of Cdk5 would also lead to increased hippocampal epileptiform activity and seizure susceptibility.

## Results

### Conditional knockout of Cdk5

To circumvent the neonatal lethality of the constitutive Cdk5 KO mice and non-specificity of pharmacological inhibitors [11], [21]–[23], conditional KO of Cdk5 was achieved by deriving mice in which loxP elements flanked critical exons in both Cdk5 alleles. Recombination was mediated with a transgenic estrogen receptor-Cre recombinase fusion, under control of the prion promoter [24] in response to a 15-day regimen of hydroxytamoxifen as previously described [7]. Hydroxytamoxifen-dosed wild-type littermate mice served as controls (WT).

### Altered theta burst synaptic response accompanies enhanced LTP after conditional knockout of Cdk5

**Figure 1:**
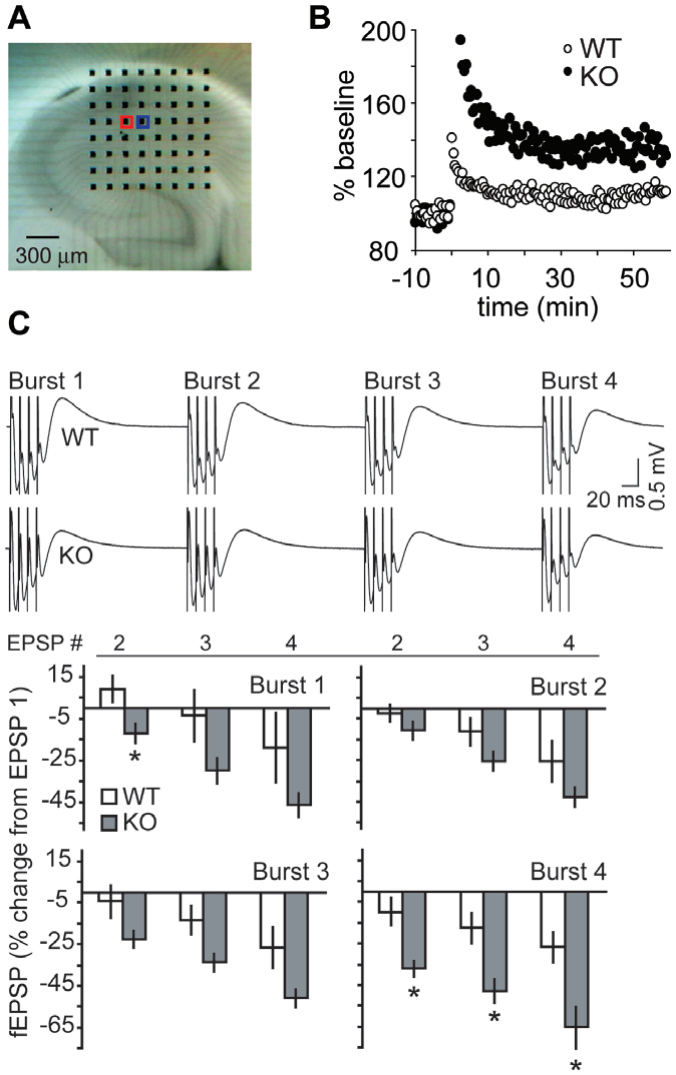
Altered theta burst topography in SC/CA1 pathway accompanies enhanced LTP in Cdk5 KO mice. *A*, An acute hippocampal slice resting on MED-64 multi-electrode array. fEPSPs were recorded (blue) after stimulation of the SC/CA1 pathway (red). *B*, LTP after a ten-burst theta stimulus in representative WT and KO slices plotted as percent amplitude of baseline (−10 to 0 min). *C*, Effects of Cdk5 KO on theta-burst responses. Representative theta burst traces were extracted from LTP experiment illustrated in *B*, Quantitation plotted as percentage change in amplitude (relative to the 1st fEPSP) of the 2^nd^, 3^rd^, and 4^th^ field EPSP within a single stimulus train of 4 pulses at 100 Hz in control and KO slices. The measures are shown for bursts 1–4 of a train. Similar results were obtained with slope calculations, n = 5–8. *P<0.05 vs. WT; *post hoc t*-test. Data represent mean±s.e.m.

LTP was induced in hippocampi from Cdk5 WT and KO animals within the SC/CA1 pathway on a 64-channel multielectrode array (**Figure 1A**). As expected, Cdk5 KO mice displayed a reduced threshold for LTP induction (**Figure 1B**) [7]. To determine if this enhancement was due to increased responsiveness during the theta burst stimulation (TBS) used to induce LTP we examined the fEPSPs during TBS. In WT slices, theta bursts briefly led to facilitation followed by moderate depression. In contrast, in KO slices, a TBS produced an immediate depression and subsequent larger magnitude depression compared to controls (**Figure 1C**). The 10 ms inter-stimulus interval produced a 8.1±6.3% facilitation in WT slices, but in KO slices we observed a 12.9±4.7% depression (**Figure 1C**; Burst 1, EPSP 2). Interestingly, this significant and notable discrepancy between WT and KO mice was specific for the shortest intervals of 10 ms (100 Hz). Paired-pulse potentiation at inter-stimulus intervals (ISI) between 25 and 800 ms (40–1.25 Hz) were not significantly different [9]. Repolarization after a theta burst stimulus is, in part, dependent on Ca^2+^-activated K^+^-channels [25]. To evaluate for possible aberrations in Ca^2+^-activated K^+^-channels, burst duration and repolarization were examined. Analysis revealed no differences between KO and WT in both burst duration and rebound during theta burst stimuli.

### Responses to CA1 hippocampal tetanic stimuli in WT and Cdk5 KO mice

**Figure 2:**
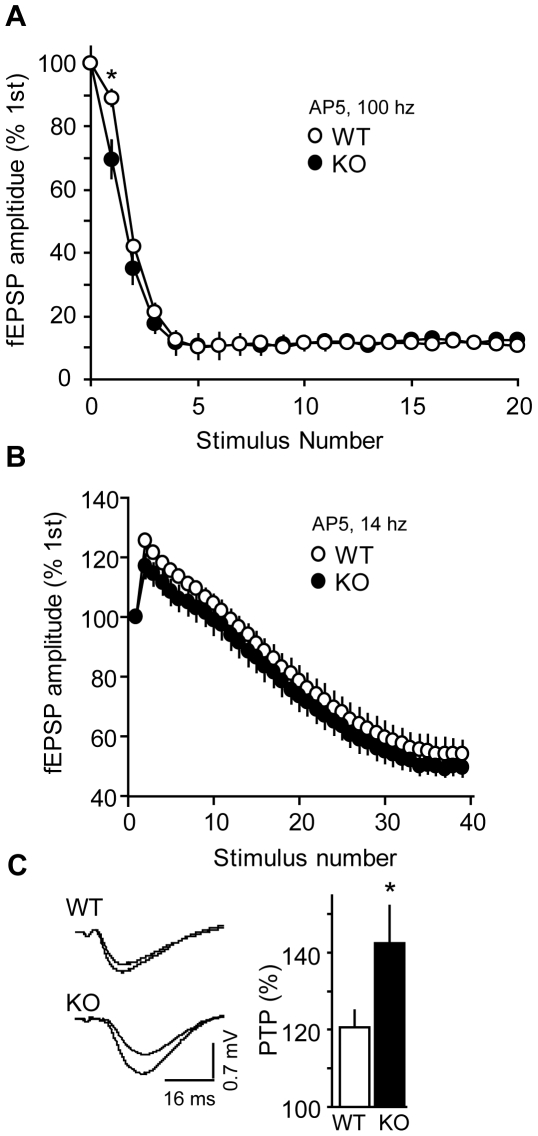
Tetanic stimulation and post-tetanic potentiation after conditional loss of Cdk5. *A*, fEPSPs during high frequency tetanus. fEPSPs amplitudes during 100 Hz tetani are plotted relative to the 1^st^ response in the presence of 75 µM AP5. Asterisk indicates that the 2^nd^ fEPSP during the train was significantly different between WT and KO (P<0.05, student's *t*-test). *B*, Effects of Cdk5 KO on fEPSPs during low frequency tetanus. fEPSPs amplitudes during 14 Hz tetani are plotted relative to the 1^st^ response in the presence of 75 µM AP5. *C*, Post-tetanic potentiation after a 100 Hz stimulus. Representative traces and fEPSP amplitudes were measured and plotted relative to baseline (75 µM AP5, n = 5–8; P<0.05, student's *t*-test). Data represent mean±s.e.m.

To further evaluate synaptic plasticity in WT versus Cdk5 KO mice, responses to high and low frequency tetani were measured. The fEPSP amplitudes were measured during a 100-Hz high frequency tetanus in the presence of NMDAR antagonism. As with the TBS in **Figure 1**, this experiment revealed a paired-pulse disparity between KO and WT (**Figure 2A**). Because this experiment was performed in the presence of a NMDAR-antagonist, the enhanced depression was not due to NMDA activation. After the 2^nd^ pulse, Cdk5 KO and WT mice displayed similar responses to 100-Hz tetani (**Figure 2A**). The fEPSPs during low frequency tetani are often measured to assess the status of the presynaptic reserve vesicle pool. A 14 Hz train in the presence of a NMDAR antagonist elicited equivalent fEPSP facilitation followed by vesicle depletion and fEPSP depression in both groups (**Figure 2B**). In the presence of NMDA antagonism these data show no detectable aberration in the presynaptic reserve vesicle pool due to Cdk5 loss.

### Conditional loss of Cdk5 leads to an enhancement in CA1 hippocampal post-tetanic potentiation

After a high frequency tetanus, neurons exhibit a form of short-term plasticity called post-tetanic potentiation (PTP). Although much of this phenomenon remains uncharacterized, PTP is likely due to a tetanus-induced elevation in presynaptic Ca^2+^ which leads to a short-lived increase in vesicle release [26]. Changes in PTP could result from alterations in presynaptic Ca^2+^ channel properties or the size of the readily releasable pool of vesicles. Conditional loss of Cdk5 led to an enhancement in PTP following a either a 100 Hz tetanus (**Figure 2C**) or TBS [7]. In the presence of a NMDAR antagonist, a 100 Hz tetanus produced 120.7±4.3% and 142.4±9.7% PTP in WT and KO slices, respectively. These findings indicate that loss of Cdk5 enhances NMDA-independent short-term plasticity.

### Conditional loss of Cdk5 leads to elevated Mg^2+^-sensitive potentials

**Figure 3:**
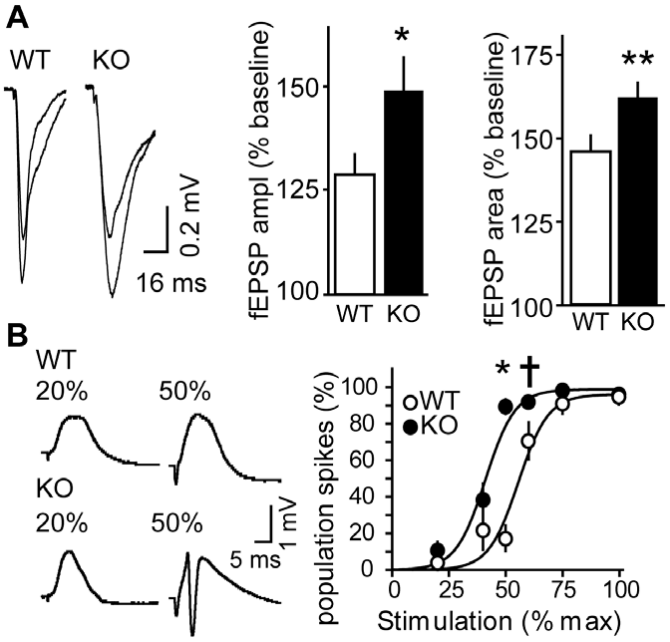
Increased Mg^2+^-sensitive post-synaptic potential and reduced threshold for epileptiform activity after conditional loss of Cdk5. *A*, Representative traces and quantitation of fEPSP amplitude (n = 12) and area (n = 6) in the *stratum radiatum* relative to baseline following Mg^2+^ wash-out. *B*, Representative traces and quantitation showing reduced threshold for population spikes in the *stratum pyramidale* of KO slices after stimulation in the hippocampal SC/CA1 pathway in Mg^2+^-free conditions. Quantitation shows % of slices displaying population spikes at indicated stimulation intensities (% of maximum). Recordings were performed 4–6 weeks post-KO. n = 16–18; *ANOVA* revealed a main effect of genotype (F_1,217_ = 36.16, p<0.0001), stimulation (F_6,217_ = 115.41, p<0.0001) and interaction between genotype and stimulation (F_6,217_ = 11.76, p<0.0001). **P<0.05, *P<0.01, ^†^P = 0.067 vs. WT, *post hoc t*-test. Data represent mean±s.e.m.

In addition to their role in LTP induction, NMDARs play key roles in many intracellular signaling cascades, neuronal excitability as well as seizure generation [27]. Consequently, *in vitro* slice physiology epileptiform activity is often measured in Mg^2+^-free conditions [28], [29]. Since loss of Cdk5 led to a NMDAR-mediated enhancement in synaptic plasticity, we analyzed Mg^2+^-sensitive potentials in Cdk5 KO mice, thus allowing measurement of fEPSPs containing a predominant NMDA component. Mg^2+^-sensitive evoked fEPSP measurements were taken within the SC/CA1 hippocampal pathway. The removal of NMDA receptor Mg^2+^-blockade produced a 1.71-fold larger fEPSP amplitude change in KO than WT (148.9±7.6 vs. 128.6±4.7% of baseline, respectively; **Figure 3A** **, left**). There was also greater overall charge transfer in Cdk5 KO mice versus controls as measured by fEPSP areas (162.3±4.4 vs. 145.2±5.7% of baseline, respectively; **Figure 3A** **, right**). These findings indicate that loss of Cdk5 led to increased Mg^2+^-sensitive post-synaptic potentials suggesting increased NMDAR function.

### Chronic loss of Cdk5 reduced threshold for epileptiform population-spike activity

Hippocampal NMDAR activation is an important step in many *in vitro* and *vivo* seizure models [27]. Abnormal neuronal excitability may produce epileptiform-like activity in acute brain slices. Hyperexcitability can be characterized by an increased propensity for populations of cells to fire in synchrony generating, so called, population spikes. We examined whether conditional loss of Cdk5 increased susceptibility to *in vitro* epileptiform activity in the SC/CA1 pathway after unmasking NMDARs. Synaptically-evoked fEPSPs in the *stratum pyramidale* were measured in Mg^2+^-free conditions. Stimulation of the SC/CA1 pathway at 20%-maximal stimulation intensity produced population spikes in 3.7±2.5% of WT and 10.4±5.0% of KO slices. A stronger 50%-maximal stimulation intensity produced population spikes in 89.6±4.0% of KO slices but only 16.74±7.7% of WT slices (**Figure 3B**). Stronger stimulation intensities elicited population spikes in greater number and larger magnitude in both KO and WT slices. These results suggest show that loss of Cdk5 reduces the threshold to induce evoked epileptiform activity in hippocampal slices.

**Figure 4:**
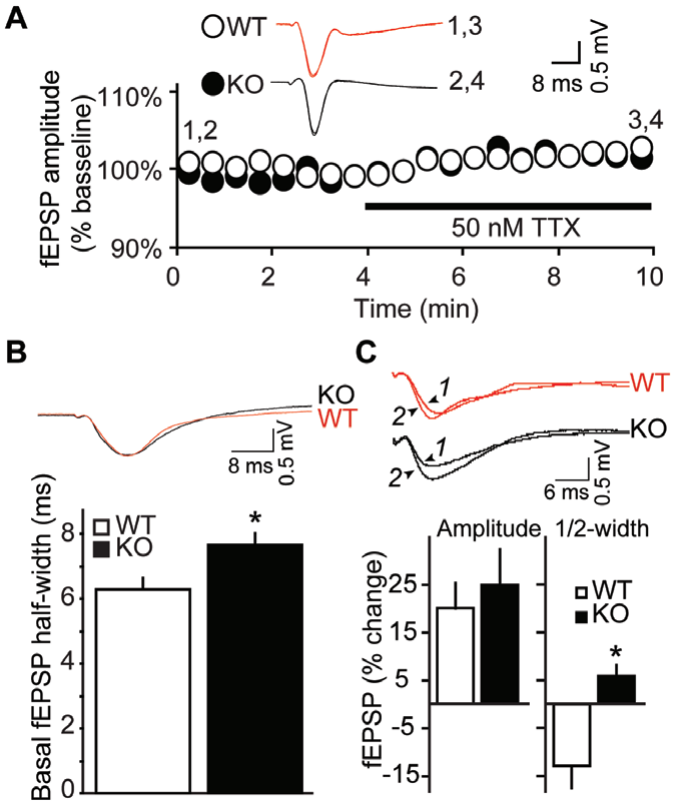
GABA_A_-mediated signaling partially compensates for increased fEPSP half-width in Cdk5 KO mice. *A*, Effect of partial Na^+^-channel blockade on fEPSP amplitude. Representative fEPSPs traces from WT (red) and KO (black) are shown before and after TTX treatment (n = 12). *B*, Increased basal fEPSP half-width in SC/CA1 pathway of KO hippocampus. Representative fEPSPs traces from WT (red) and KO (black) are shown with quantitation (n = 9–10). *C*, Representative fEPSPs traces before and after GABA_A_ blockade (top) are shown with quantitation (bottom). Histograms show the changes in amplitude and half-width following treatment with 2 µM SR95531 (n = 7). *1* and *2* indicate traces before and after treatment with SR95531, respectively. SR95531 had similar effects on fEPSP slope measurement. *P<0.05 vs. WT; *post hoc t*-test.

Aberrations in Na^+^ channel properties can affect neuronal excitability [30]–[33] and Na^+^ channel antagonists serve as a therapeutic anticonvulsants. Basal population-spike thresholds are dependent on Na^+^ channel activation. Therefore, we surveyed the basal *in vitro* population-spike threshold and the effects of partial Na^+^ channel blockade on fEPSPs. To analyze population spike threshold, input/output measurements were collected in the *strata radiatum* and *pyramidale*. The stimulation thresholds to induce population spikes in both layers were equivalent between groups. Furthermore, partial blockade (>50%) of Na^+^ channels with 50 nM TTX produced no changes from baseline in fiber volley or fEPSP amplitudes in KO and WT groups (**Figure 4A**). Loss of Cdk5 lowered the threshold for population spikes and this could not be rescued by substantial reduction (>50%) in Na^+^ channel availability [34].

Although the increase in NMDAR-mediated current in Cdk5 KO mice [7] likely contributes to the neuronal hyperexcitability, additional mechanisms are also possible. Statistical analysis of basal synaptic responses in the SC/CA1 *stratum radiatum* layer in Mg^+^-containing conditions revealed that Cdk5 KO mice, 2–4 weeks post-KO, had significantly longer fEPSP half-widths compared to WT mice (7.7±0.4 vs. 6.3±0.4 ms, respectively; **Figure 4B**). This increase in fEPSP duration likely represents an impairment in repolarization and may contribute to increased neuronal excitability. Antagonism of NMDARs, Ca^2+^ channels, and Na^+^ channels did not reverse the elevated fEPSP half-width in Cdk5 KOs (not shown). Impairments in inhibitory signaling could result in behavioral hyperexcitability [27], [35], [36]. Therefore, the effects of GABA_A_ channel antagonists on fEPSPs were measured. Administration of SR95531 led to equivalent increases in fEPSP amplitudes in WT and KO (20.1±5.2 vs. 25.1±12.1% increase from baseline, respectively; **Figure 4C**, left). However, SR95531 decreased the fEPSP half-width in WT but not KO slices (−12.8±5.9% vs. +5.9±1.7% change, respectively; **Figure 4C** right). These results suggest that GABA_A_-mediated signaling in Cdk5 KO mice may compensate for the increased fEPSP duration.

### Increased behavioral seizure susceptibility follows chronic loss of Cdk5

**Figure 5:**
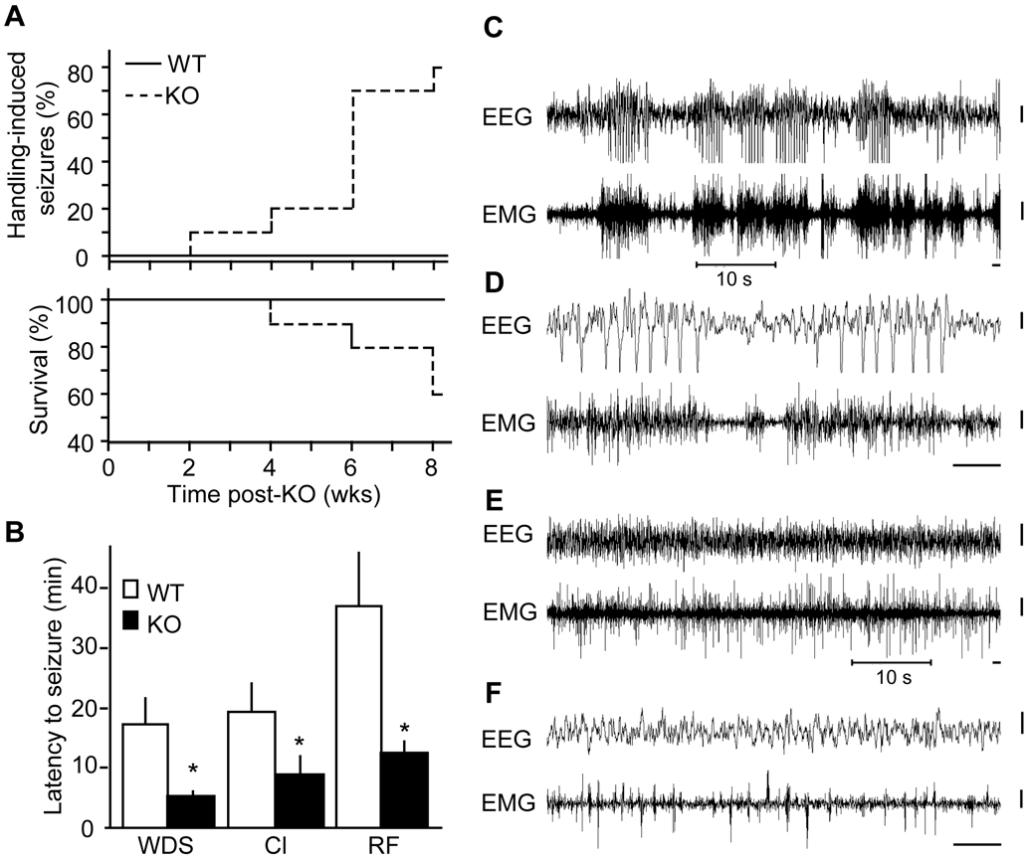
Elevated seizure susceptibility after chronic conditional Cdk5 loss. *A*, Kaplan-Meier seizure morbidity and survival curves. Percent of animals observed to have handling-induced seizures and percent survival are plotted against weeks post-KO; n = 10–14; the differences for morbidity and mortality were statistically significant, P<0.01, log-rank test. *B*, Latency to pilocarpine-induced wet-dog shakes (WDS), clonus (Cl), and rearing/falling (RF) seizures in mice, 8 weeks post-KO (n = 7–10). *P<0.05 vs. WT, Student's *t-*test. Data represent means±s.e.m. *C–F*, Representative spontaneous EEG/EMG recordings from a conditional KO mouse, 8 weeks post-KO. *C*, Appearance of multiple bursts (at 3–4 Hz) of spike waves which were frequently clustered as shown and accompanied by evident tremor and clonic seizures. *D*, Expanded view of 10 sec of the recording period shown in Figure *C*, displaying two spike bursts separated by about 2 sec. *E*, Post-ictally, there was rapid return to normal EEG/EMG and resumption of ongoing activity as displayed here during a period of wakefulness. *F*, Expanded view of 10 sec of the recording period shown in Figure *E*, Calibration 1 sec and 50 µV; the recording periods shown in panels *D* and *F* are annotated by the 10 sec bars in *C* and *E* respectively.

Although NMDARs are critical for synaptic plasticity and learning, too much NMDAR activity may be harmful [37]. During our studies, we found that prolonged loss of Cdk5 was associated with handling induced seizures and lethality when compared with controls. This observation prompted quantitative assessment of susceptibilities to handling-, pharmacologically-, and audiogenically-induced seizures. Hydroxytamoxifen-dosed *Cre* negative mice (WT) and vehicle-dosed animals carrying the *Cre* transgene alone displayed no spontaneous, handling-induced, or audiogenically-induced seizures. Furthermore, pharmacologically-induced behavioral seizure susceptibilities and latencies were equivalent between the vehicle-dosed and WT groups (not shown). On the other hand, chronic loss of Cdk5 led to handling-induced head nodding, wet dog shakes, or clonus behavioral seizures in 80% of mice after 8 weeks of Cdk5 KO (**Figure 5A**, top; **Table 1**). While mild handling induced Racine scale 1–3 class seizures, more stressful conditions, such as water immersion, triggered Racine scale 3–5 seizures, tonus and death in additional cohorts of chronic KO mice. Interestingly, increased seizure susceptibility correlated with lethality as loss of Cdk5 for 8 weeks and daily handling resulted in 40% mortality (**Figure 5A**, bottom; **Table 1**). In summary, chronic loss of Cdk5 led to increases susceptibility to handling-induced behavioral seizures and increases lethality.

**Table 1:** Long-term conditional Cdk5 knockout increases susceptibility to seizures.

Category of seizures	genotype	Morbidity	Seizure latency (wks^1^)	Mortality^2^
Handling-induced	WT	0% (0/12)	n/a	0% (0/12)
	KO	80% (8/10)	5.63±0.53	40% (4/10)
Category of seizures	genotype	Clonus	Status epilepticus	Tonus & Death
Chemically-induced^3^	WT	71% (5/7)	57% (4/7)	42% (3/7)
	KO	100% (7/7)	100% (7/7)	86% (6/7)

1 Weeks after last dose of hydroxytamoxifen.

2 Within 8 wks.

3 325 mg/kg pilocarpine, i.p./2.5 mg/kg scopolamine, *s.c.*

To evaluate and quantify seizure susceptibility in Cdk5 KO mice, seizure latencies were scored after animals were treated with a convulsant, pilocarpine. Cdk5 KO mice displayed increased susceptibility (**Table 1**) and significantly shorter latencies than WT to undergo wet dog shakes (5.4±0.6 vs. 17.3±4.3 min, respectively), clonus (9.0±3.0 vs. 19.3±4.8 min), and rearing and falling (12.53±1.9 vs. 37.0±12.3 min; **Figure 5B**). Hence, Cdk5 KO led to 68.7%, 53.7%, and 66.2% decreases in mean latencies to induce wet dog shakes, clonus, and rearing and falling behavioral seizures. Chronic loss of Cdk5 led to increase central nervous system excitability exhibited by the decreased threshold for chemically-induced seizures.

Cranial electronencephalographic (EEG) electrodes and electromyographic (EMG) electrodes were implanted into mice and spontaneous activity was recorded for characterization of electrographic activity in WT versus Cdk5 KO mice. After loss of Cdk5 for 8 weeks, KO mice displayed epochs of multiple bursts (at 3–4 Hz) of spike waves which correlated with tremor activity on EMG (**Figure 5C–D**). Post- and inter-ictally, there were normal EEG/EMG activities during wakefulness and sleep (**Figure 5E–F**). Seizure activity was not observed in littermate hydroxytamoxifen-dosed WT controls (**Figure S1**). In summary, loss of Cdk5 for 2 months led to increased lethality, elevated seizure susceptibility, and electrographic evidence of seizure activity.

### Increased acoustic startle reactivity in Cdk5 KO mice

**Figure 6:**
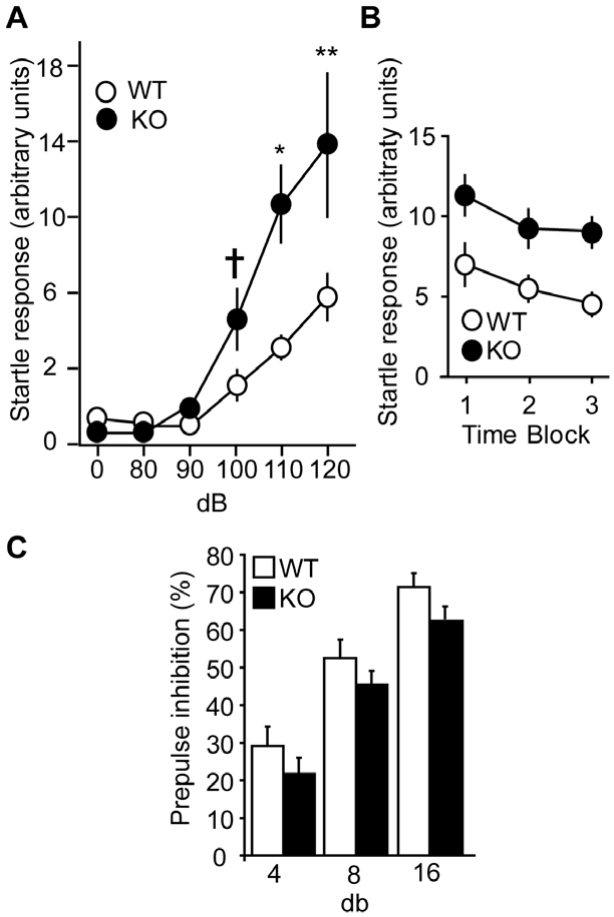
Increased acoustic startle reactivity with normal short-term acoustic habituation and prepulse inhibition in Cdk5 KO mice. *A*, Acoustic startle reactivity in Cdk5 KO mice (4–8 weeks post-KO, n = 17–19). *ANOVA* revealed a main effect of genotype (F_1,204_ = 16.35, p<0.0001), decibels (F_5,204_ = 19.67, p<0.0001) and interaction between genotype and decibels (F_5,204_ = 3.72, p = 0.003). *P<0.01, **P<0.001, ^†^P = 0.08 vs. WT, *post hoc t*-test. *B*, Habituation to a 120 db sound is shown as startle reactivity in three consecutive time blocks. *ANOVA* revealed effects of genotype (F_1,68_ = 8.62, p<0.006) and trial (F_2,68_ = 14.65, p<0.0001) but no interaction between genotype and trial (F_2,68_ = 0.31, p = 0.7326). n = 17–19. *C*, Prepulse inhibition in Cdk5 KO mice. Prepulse at indicated decibels were given 5 ms prior to a 120 db sound. Prepulse inhibition is represented as percent decrease compared to an isolated 120 db sound. *ANOVA* revealed effect of decibel (F_2,32_ = 143.74, p<0.0001) but no effect of genotype (F_1,32_ = 2.03, p = 0.1735) or interaction (F_2,32_ = 0.78, p = 0.469). n = 8–10. Vehicle-treated animals behaved similarly to WT. Data represent mean±s.e.m.

As an additional study of behavioral excitability, the brainstem primary acoustic startle circuit was examined by measuring acoustic startle reactivity. Animals were exposed to auditory stimuli (white noise) at various decibels and startle responses were measured. Cdk5 KO startle responses were 1.8±0.6-, 2.2±0.5-, 2.5±0.4-, and 2.3±0.6-fold larger than WT after 90, 100, 110, and 120 db pulses, respectively (**Figure 6A**). No differences between WT and KO were observed in habituation of startle responses (**Figure 6B**) or pre-pulse acoustic inhibition (**Figure 6C**). Thus, loss of Cdk5 in the adult brain elevated the acoustic startle responses but had no detectable difference on short-term acoustic habituation or pre-pulse inhibition.

### Audiogenic behavioral seizures after chronic loss of Cdk5

Audiogenic stimulation rarely produces seizures in healthy wild types animals. However, in special animal breeds, after kindling, or with certain genetic modifications, high-frequency acoustic tones can produce behavioral and electrographic seizure activity. While chemically-induced seizures are generally thought to model seizures of hippocampal or temporal lobe origin, audiogenic seizures may originate in the brainstem [29]. Given that loss of Cdk5 led to increases in acoustic startle reactivity, we examined whether loss of Cdk5 reduced the susceptibility to audiogenic seizures. Short-term loss of Cdk5 (2–4 weeks) did not increase susceptibility to audiogenic behavioral seizures (0/10 short-term Cdk5 KO animals exhibited behavioral seizure activity). In contrast, prolonged loss of Cdk5 (for 8 weeks) increased susceptibility to audiogenically-induced seizures. High frequency audiogenic stimulation had no effect on WT but induced wild running in 37% of KO mice in 32.3 sec (**Table 2**). Wild running progressed to tonic-clonic seizures and tonus followed by death in 18% of KO animals within 56.0 sec. Consequently, chronic loss of Cdk5 in the adult brain increased the propensity towards acoustically-induced behavioral seizures.

**Table 2:** Audiogenic seizures after long-term conditional Cdk5 knockout^1^.

Genotype	Wild Running		Tonic-clonic seizure		Tonus/death	
	Morbidity	Latency (s)	Morbidity	Latency (s)	Morbidity	Latency (s)
WT	0% (0/10)	n/a	0% (0/10)	n/a	0% (0/10)	n/a
KO	27% (3/11)	32.3±6.7	18% (2/11)	50.0±11.0	18% (2/11)	56.0±6.0

1 After exposure to 90 db, 2.8 kHz pulse tone for 3 min

### Status epilepticus and electroconvulsive shock leads to the production of Cdk5-activating cofactor in the hippocampus

**Figure 7:**
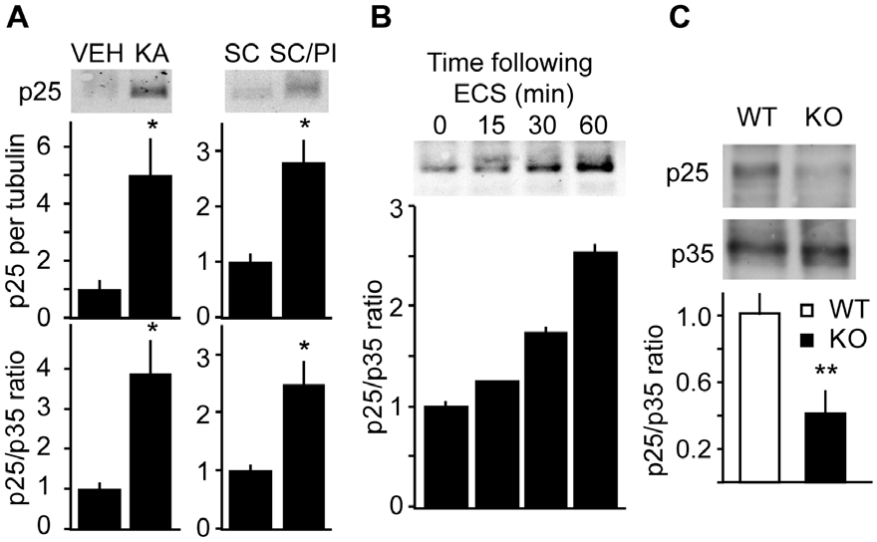
p25 generation in the hippocampus after pharmacologically-induced status epilepticus and electroconvulsive shock. *A*, Representative immunoblots (top) and quantitation of p25 per tubulin (middle) and p25/35 ratio (bottom) after treatment with kainate and pilocarpine. VEH = saline; KA = kainate (50 mg/kg); SC = scopolamine (2 mg/kg)/saline; and SC/PI = scopolamine (2 mg/kg)/pilocarpine (280 mg/kg). n = 4; *P<0.05 vs. VEH and SC in; Student's *t-*test. *B*, Representative immunoblots of p25 and quantitation of p25/p35 ratio 15, 30, and 60 min following electroconvulsive shock administration compared to unshocked controls (time = 0); n = 2. *C*, Hippocampal p25∶p35 ratio 6–8 weeks post-KO. Crude lysates were immunoblotted for p25 and p35. Double asterisk indicates P<0.05 versus aged WT, Student's t-test; n = 3–4. Data represent means±s.e.m.

Both conditional KO of Cdk5 [7] and transient overexpression of p25 [5] result in increased synaptic plasticity and learning. There may be common mechanistic features between loss of Cdk5 and diversion of Cdk5 to form a complex with p25. Interestingly, human hippocampal sclerosis is accompanied by p25 generation and altered Cdk5 activity [38]. To further assess the role of Cdk5 in acute seizures and the homeostasis of neuronal excitability, status epilepticus was induced pharmacologically in WT mice and p25 levels were assessed in hippocampal lysates. Kainate-induced status epilepticus led to 5.0±2.7- and 3.9±1.3-fold increases in p25 level and p25/p35 ratio, respectively (**Figure 7A**, left). Similarly, pilocarpine-induced status epilepticus caused 2.8±0.8- and 2.5±0.6-fold increases in p25 level and p25/p35 ratio (**Figure 7A**, right). Hence, chemically-induced status epilepticus in normal WT animals caused p25 levels in increase in hippocampal lysates.

Chemically-induced acute seizures only represents one of many models used to study seizures. Generation of p25 was also analyzed following electrical electroconvulsive shock. Electroconvulsive shock, which causes broad forebrain epileptiform activity, is clinically used to treat depression [39] and experimentally used for the generation of seizures [40]. Acute electroconvulsive shock resulted in time-dependent accumulation in p25 in hippocampus (**Figure 7B**). Sixty minutes following electroconvulsive shock, p25 levels increased 2.47±0.08-fold. The findings suggest that both pharmacologically- and electrically-induced epileptiform activity results in accumulation of the Cdk5 activating cofactor, p25.

### Chronic but not acute loss of Cdk5 leads to reduced levels of Cdk5-activating cofactor in the hippocampus

Studies implicating Cdk5 and p25 in neuronal excitability suggest that Cdk5 may be activated in order to either attenuate or modulate excitability during seizures. Since Cdk5 KO mice displayed elevated neuronal excitability and susceptibility to seizures, we examined the levels of p25 in Cdk5 KO mice. Hippocampi from subacute Cdk5 KO mice (2–4 weeks after KO) contained similar levels of p25 compared with controls (111.7±13.7% of WT, p = 0.6). However, chronic loss of Cdk5 for 6–8 weeks led to a 58.4±12.4% reduction in basal levels of p25 (**Figure 7C**). Consequently, chronic but not acute loss of Cdk5 leads to reduced levels of p25. The decrease in p25 levels corresponds to an increase in neuronal excitability and seizure susceptibility.

## Discussion

Previous studies have implicated Cdk5 in the regulation of neuronal excitability [1]. Cdk5 has been suggested to both increase [41], [42] and decrease [43] neurotransmitter release and modulate striatal neuron excitability [1]. Cdk5 has specifically been implicated in exocytosis and endocytosis via phosphorylation of numerous substrates including synapsin, amphiphysin I, dynamin, and others [44]–[47]. We previously demonstrated that conditional loss of Cdk5 led to enhancements in plasticity and learning via modulation of NMDAR degradation [9]. Furthermore, transient overexpression of Cdk5-activating cofactor, p25, increases NMDAR-mediated plasticity and synaptic transmission [7], [15].

Here, we further characterize the enhancement of hippocampal plasticity in Cdk5 KO mice by studying fEPSPs during theta bursts and tetanic stimuli. We first show that within the SC/CA1 pathway, Cdk5 KO mice displayed altered LTP-inducing TBS topography. During the detailed LTP analyses, we find that loss of Cdk5 also leads to an NMDAR-independent enhancement in PTP, a presynaptic form of short-term plasticity. Although Cdk5 KO had no effect on paired pulse facilitation (PPF) with interstimulus intervals between 25 and 800 [7], Cdk5 KO did lead to depression with a shorter 10 ms inter-stimulus interval. Cdk5 may theoretically confer these changes by increasing the number of presynaptic vesicles, increasing presynaptic Ca^2+^-influx, or increasing sensitivity to presynaptic Ca^2+^. Changes in probability of presynaptic vesicle release are usually accompanied by broad impairments in PPF. However, since loss of Cdk5 only conferred a PPF deficit at a very short interstimulus interval, additional information is necessary to better elucidate Cdk5′s role in the presynapse. Although several studies implicate Cdk5 in vesicle release and recycling [41]–[43], [48], the exact nature of Cdk5′s role in the presynaptic compartment is still unclear. Future studies examining Cdk5′s role in presynaptic terminal would be beneficial.

Conditional loss of Cdk5 initially leads to enhanced learning, plasticity and increased NMDAR-mediated currents [7]. In the present study, electrophysiological extracellular hippocampal recordings *in vitro* reveal that conditional loss of Cdk5 also leads to elevations in fEPSPs with a predominant NMDA component and reduced threshold for population-spike activity. It is known that the NR2B NMDAR subunit directly modulates neuronal excitability and contributes to seizures [49]–[52], so it is possible that a similar mechanism is involved following loss of Cdk5. Other mechanisms may also contribute to the enhanced hippocampal excitability. For example, loss of Cdk5 led to a subtle impairment in fEPSP repolarization in the hippocampal SC/CA1 pathway. Nonetheless, the data suggests that Cdk5 functions to attenuate hippocampal neuronal excitability via several mechanisms including the modulation of NMDARs.

Over time, Cdk5 KO mice displayed an increase in seizure susceptibility, suggesting a progressive increase in excitability. Chronic loss of Cdk5 increased the propensity for pharmacologically- and audiogenically-induced seizures. The reduced threshold for behavioral seizure activity correlated with EEG/EMG evidence of spontaneous seizures. Given the complexity, impact, and significance of spontaneous seizure activity, additional EEG recordings over an extended period of are warranted in future studies. In addition, conditional Cdk5 KO mice also displayed an increase in behavioral startle reactivity. We also found an association between the seizure phenotype and the lethality in Cdk5 KO mice: after 10 weeks of Cdk5 KO, the majority of Cdk5 KO mice displayed signs of increased behavioral excitability and propensity towards handling-induced seizures. Up to 40% of affected mice died soon after displaying seizure activity.

The electrophysiological and behavioral experiments reveal similar trends. Initially, Cdk5 KO mice display increased NMDAR-mediated currents, enhanced plasticity, and impaired neuronal repolarization. Later, mice exhibit increased startle reactivity and reduced threshold for *in vitro* population spikes. Then, mice exhibit spontaneous electrographic seizures, handling-induced seizures, audiogenic seizures and lower threshold for pharmacologically-induced seizures. Although the experiments were performed at varying time-points after Cdk5 KO, together, the results suggest that loss of Cdk5 leads to a progressive increase in neuronal and behavioral excitability, ultimately leading to seizures.

Abnormal expression and dysregulation of Cdk5 and its cofactors have been demonstrated in tissues from human cortical dysplasia [53] and hippocampal sclerosis [38], [46], [54]. KO of p35 leads to cortical lamination defects, seizures, and lethality [55]–[57]. Mice lacking p35 also display abnormal morphological and functional organization of the hippocampus, dysplastic hippocampi, heterotopic pyramidal cells, and granule cell dispersion and may serve as a model for cortical dysplasia [29], [57]. In this study we show that chemically-induced status epilepticus and electroconvulsive shock in healthy animals induced acute generation of a Cdk5-activating cofactor, p25. Chronic loss of Cdk5 is associated with both seizures and reduced levels of p25. These published and new findings indicate that Cdk5 and its cofactors may play key regulatory roles in neuronal excitability.

Recent data have demonstrated dual roles for both Cdk5 and its activating cofactor, p25, in learning and plasticity [5], [7]. A dichotomy may also exist for Cdk5/p25′s role in pathological neuronal excitability associated with seizures. Loss of Cdk5 increases excitability and leads to seizures, which corresponds with decreased levels of p25. Acute seizures in healthy animals and chronic seizures in human epileptics produces elevated levels p25 [54]. During periods of increased Ca^2+^-influx, neurons produce p25 following calpain activation [58]. Initially, Cdk5/p25 may serve as a homeostatic molecule to dampen excitatory transmission and inhibit seizure activity. However, over-excitation could result in excessive Ca^2+^-influx and p25 generation, aberrant Cdk5 activity and neurotoxicity [59]. Thus, Cdk5 may serve to inhibit abnormal epileptiform activity when a neuron is in a normal state or promote cell death following excess and non-physiological Ca^2+^ influx. We previously showed that Cdk5 facilitates calpain-mediated degradation of the NR2B NMDAR subunit [7]. Cdk5 KO may impair calpain-mediated p25 generation and thereby disrupt normal homeostatic mechanisms that prevent seizures. Future studies on Cdk5, calpain, p25, and NR2B may better our understanding of the divergent roles for Cdk5 in neuronal physiology and disease.

Current anticonvulsant therapeutics increase inhibitory neurotransmission, suppress high frequency neuronal firing by reducing voltage-gated Na^+^ channels availability, or inhibit voltage-gated T-type Ca^2+^-channels. Such therapeutic options produce unwanted side effects, are only efficacious in 70% of adults suffering from recurrent seizures [60], and generally alleviate the symptoms rather than curing or modifying the underlying etiology [27]. Aberrations in neuronal excitability can produce abnormal electrical discharges in the brain leading to seizure activity. Thus, a better understanding of the cellular mechanisms underlying neuronal excitability will aid in the development of novel therapeutics for seizures.

In future studies, it will be worthwhile to directly assess whether the increase in neuronal excitability following Cdk5 KO has any affects on presynaptic transmission, neuronal fiber sprouting, cell count, and cell survival. Furthermore, it would be interesting to study how long-term loss of Cdk5 affects hippocampal plasticity and learning. Although short-term loss of Cdk5 produced enhancements in plasticity and learning [7], it is possible that chronic loss of Cdk5 and the associated epileptiform activity leads to neurodegeneration and impaired synaptogenesis, learning, and structural plasticity [5], [16].

## Materials and Methods

### Animals and reagents

All procedures have been conducted in accordance with relevant NIH, national and international guidelines and were approved by the UT Southwestern Institutional Animal Care and Use Committees. Unless otherwise noted, mice were housed 4 per cage in a colony maintained at 23°C with a 12 h light/dark cycle (lights on from 7:00 A.M. to 7:00 P.M.) and *ad libitum* food and water. Breeding, genotyping, and KO induction were performed as previously described [7]. Adult mice were treated with 4-hydroxytamoxifen for 15 days (66.67 mg per kg, *i.p.*). Post-KO time period refers to the time following end of hydroxytamoxifen treatment. Hydroxytamoxifen-dosed mice with *floxed Cdk5* alleles mice served as WT littermate controls, as previously reported [7]. As additional controls, electrophysiolgical and behavioral experiments were also performed on genetic control strains: vehicle-dosed and undosed mice with and without *Cre* transgene and *floxed Cdk5* alleles. All experiments were performed on adult male mice with experimenter blind to genotype. Unless otherwise specified, reagents were purchased from Sigma.

### Electrophysiological recordings

Transverse hippocampal slices from 8–14 week old males were prepared in cutting saline (200 mM sucrose, 3 mM KCl, 1.4 mM NaH_2_PO_4_, 26 mM NaHCO_3_, 2 mM MgCl_2_, 2 mM CaCl_2_, 10 mM glucose) and maintained in an interface chamber containing artificial cerebrospinal fluid [61], [62]. Extracellular field excitatory post-synpatic potentials (fEPSPs) in the Schaffer Collateral/CA1 hippocampal pathway (SC/CA1) were synaptically evoked at 0.033 Hz and recorded in the *stratum pyramidale* and *stratum radiatum* layers using a 64-channel array (150 µm interpolar distance, MED-P5155, Alpha MED Sciences). Input-output measurements were performed as described [7] and, unless otherwise stated, fEPSPs were evoked using a stimulation intensity which elicited 50% maximal response. Data acquisition and analysis were performed using the Multielectrode MED64 hardware and software packages (Panasonic) essentially as described [7]. Stimulus artifacts were removed and additional analyses were performed using custom macros running under Igor Pro, Microsoft Excel, and Graphpad Prism. LTP experiments were performed as described [7]. Theta burst topography was assessed 2–4 weeks post Cdk5 KO as previously described [7], [63]. fEPSP amplitudes in the *stratum radiatum* layer were measured with respect to the first fEPSP within each theta burst.

### Post-tetanic potentiation

Post-tetanic potentiation was assessed as previously described, 2–4 weeks post-KO [7]. Basal input/output measurements were performed to determine stimulus intensity to elicit 40% of the maximal fEPSP amplitude. Input/output analysis, paired-pulse facilitation, and tetani were performed in the absence of any drugs unless otherwise indicated. Post-tetanic potentiation (PTP) was elicited in the presence of 75 µM AP5. Baseline was followed by a brief 100 Hz tetanus [23] and post-tetanus recordings. PTP measurements were made on the initial recording after the tetanus. Measurements of fEPSP slope showed similar results.

### fEPSP measurements in Mg^2+^-free conditions and epileptiform activity

Mg^2+^-free fEPSP and epileptiform activity experiments were performed 4–8 weeks post-KO. fEPSP input/output measurements were made in regular ACSF to determine the stimulation intensities required for 20% and 100% of maximal fEPSP amplitudes. Baseline and Mg^2+^-sensitive fEPSP amplitudes and areas were measured in regular ACSF and Mg^2+^-free ACSF, respectively. Mg^2+^-sensitive fEPSP measurements in the *stratum radiatum* were recorded using the 20%-maximal stimulation intensity, which allowed for accurate measurement of fEPSP magnitude in the absence of population spikes and epileptiform activity. Any fEPSP measurements contaminated with population spikes were excluded from calculations. Evoked population spikes and epileptiform activity in the *stratum pyramidale* were recorded in Mg^2+^-free buffer. Input/output measurements were recorded in Mg^2+^-free buffer in triplicate and analyzed for population spikes. Fiber volley amplitude versus stimulus intensity was not significantly changed after washout of Mg^2+^. Maximum stimulation intensities were pre-determined by measuring the stimulus and fiber volley magnitudes necessary produced the maximum fEPSP magnitudes during basal input/output measurements. Stimulation intensities were binned and data was fit with a sigmoidal curve with R^2^>95%.

Gross Na^+^ channel function was assessed by calculating population spike threshold and measuring the effect of partial Na^+^ channel blockade in normal ACSF. Traditional input/output measurement were performed in regular Mg^2+^-containing ACSF and visually analyzed for presence of population spikes in *stratum radiatum* and *stratum pyramidale*. Pecentage of slices displaying population spikes versus fiber volley amplitude was plotted. After input/output measurements, a stable baseline was recorded at 20%-maximal stimulation. fEPSP magnitudes were measured after addition of 50 nM TTX. fEPSP amplitudes were plotted relative to baseline.

### fEPSP repolarization

Repolarization experiments were performed 2–4 weeks post-KO. Half-width was measured as the duration of fEPSP at the half-maximal amplitude in normal Mg^2+^-containing ACSF. To examine contributions of Na^+^ channel, NMDAR, voltage-gated Ca^2+^ channel, GABA_A_R-mediated synaptic transmission on fEPSP amplitudes, slopes, and half-widths, fEPSPs were measured before and after treatment with 50 nM tetrodotoxin (TTX), 75 µM AP5, 0.1 or 0.5 mM NiCl, and 2 µM SR95531, respectively.

### Electronencephalographic (EEG)-Electromyographic (EMG) recordings

Adult male mice were surgically implanted for long-term EEG/EMG monitoring as previously described [64]. Mice were anesthetized with a mixture containing 25 mg/ml ketamine and 2.5 mg/ml xylazine (administered i.p. at a dose of 0.1 mL/mouse). They were then held in a stereotaxic frame fitted with a mouse adaptor (David Kopf Instruments, Tujunga, CA). The cranium was exposed, cleaned of all connective tissue, and 4 burr holes were drilled, anterior and posterior to bregma, bilaterally (AP 1.1, ML±1.45 and AP −3.5, ML±1.45). A prefabricated implant, with 4 EEG and 2 EMG electrodes, was then stereotaxically lowered and cemented to the skull using glass ionomer dental cement (Ketac-Cem Aplicap; ESPE, Norristown, PA). EMG wire electrodes were inserted contralaterally into the nuchal musculature using blunt dissection techniques. After suturing, the mouse was removed from the stereotaxic frame and allowed to recover from anesthesia. This design for the EEG/EMG implant allowed precise insertion of electrodes, targeting the frontal and occipital cortices at a consistent depth, just touching the dura, while minimizing surgical trauma.

EEG/EMG measurements were performed 8 weeks post-KO. Mice were housed individually under a 12 h light-dark cycle at 24±1°C, with standard laboratory chow (Harlan Teklad, Madison, WI) and water being replenished as necessary each day. Mice were not otherwise disturbed. They were habituated to these conditions for a week before EEG/EMG signals were recorded over 24 hour duration. Connections were made from the implanted cranial electrodes to the amplifier (Grass Model 78; Grass Instruments, West Warwick, RI) using a flexible, freely moving, lightweight cable. Amplified and filtered (EEG: 0.3–100 Hz; EMG: 30–300 Hz) signals were digitized at a sampling rate of 250 Hz, displayed using custom polygraph software, and archived for subsequent off-line analysis. Subsequently, the EEG/EMG record was visually screened for seizure epochs.

### Seizures and startle response behaviors

All behavioral experiments were performed with two groups of 8–14 week old males. The short-term KO group consisted of animals 2–4 weeks after Cdk5 loss (*i.e.*, 2–4 weeks following last does of hydroxytamoxifen). The chronic KO group consisted of animals 8 weeks following Cdk5 loss. Behavioral seizures were scored based on the Racine scale [29], [65], [66]. Seizure classes one through five were scored as mouth movements, head nodding or wet dog shakes, clonus, rearing, and rearing/falling, respectively. Behavioral seizure parameters such as wild running, tonic-clonic seizures, and tonus were also scored. To assess handling-induced seizures, animals which experienced daily handling were initially observed for signs of spontaneous seizures in home cage for 30 min and then assessed for 30 min after tail lift and scruffing. Handling-induced seizures typically included head nodding, clonus, or clonus with loss of balance. Chemically induced seizures were studied in mice 8-weeks post-KO. Animals were given scopolamine (2.75 mg/kg, *s.c*.) 5 min prior to pilocarpine (325 mg/kg, *i.p.*). Latency to and frequency of wet-dog shakes, clonus, tonus, rearing and falling and other seizures classes were recorded.

Startle response behaviors were studied 4–8 weeks post-KO using the SR-Lab system (San Diego Instruments) as described [23], [67]–[69]. Audiogenic seizures were studied 2–4 weeks and 8 weeks post-KO and induced in a plexiglass shock box with clear front and rear walls (MedAssociates). Mice were scored for baseline behavior for 3 min and scored for seizure activity during exposure to a 90 db, 2,800 Hz pulse tone for 3 min. Frequency of and latency to wild running, tonic-clonic seizures, tonus, other seizure stages, and death were recorded.

### Pharmacologically-induced status epilepticus and electroconvulsive shock

Six-week old C57BL/6 (Charles River Labs) wild-type mice were injected with vehicle (saline, *i.p.*), kainate (50 mg/kg, *i.p.*; Tocris), scopolamine (2 mg/kg, *s.c.*)/saline (*i.p.*), or scopolamine (2 mg/kg, *s.c.*)/pilocarpine (280 mg/kg, *i.p.*). Scopolamine was injected 5 min prior to pilocarpine. Animals were scored for latencies to and frequencies of seizure stages including head nodding, wet dog shakes, forelimb clonus, tonic-clonic seizures, rearing, and, rearing/falling. Hippocampi were dissected 25 min after first robust episode of rearing/falling, flash frozen, and stored at minus 80°C.

Electroconvulsive shock (ECS) was administered to adult male Sprague–Dawley rats (175–200 g) by delivering a current of 50 mA for 0.3 sec via bilateral ear clips essentially as described [70]. Hippocampi were dissected at the specified time-points after stimulation, flash frozen, and stored at minus 80°C. Animals which did not receive the shock served as controls.

### Immunoblot analysis

Frozen samples were sonicated in boiling 1% SDS containing 50 mM NaF and boiled for an additional 5 min. Protein concentrations were determined by the BCA protein assay (Pierce) using bovine serum albumin standards. An equal amount of total protein (100 µg) from each sample was subjected to SDS-PAGE followed by electrophoretic transfer to nitrocellulose membranes (Whatman). The membranes were immunoblotted using antibodies for p35/p25 (1∶200; C-19 Santa Cruz) and alpha-tubulin (1∶5000), followed by incubation with a horseradish peroxidase-conjugated anti-rabbit or anti-mouse secondary antibody (1∶8000; Chemicon). Antibody binding was detected by autoradiography using the enhanced chemiluminescence immunoblotting detection system (Amersham Biosciences) and quantified by densitometry using Image J software (NIH).

### Statistical analysis

All data was represented at mean±s.e.m. Errors for fold changes were calculated using standard error propagation rules. Differences between data groups were evaluated for significance using analysis of variance (*ANOVA*) with *post hoc t*-tests and statistical significance was set to p<0.05.

## Supporting Information

Figure S1 Representative EEG/EMG recordings from a wild-type control mouse. A, A period of normal wakefulness. B, Expanded view of 10 sec of the recording period shown in A. Calibration 1 sec and 50 µV; the recording period shown in panel B is annotated by the 10 sec bar in A.
(0.19 MB TIF)
