# Supplementary figures and images for: Regulation of Hippocampal and Behavioral Excitability by Cyclin-Dependent Kinase 5
Source: PLoS One. 2009 Jun 4;4(6):e5808. doi: 10.1371/journal.pone.0005808 (PMC2695674; doi:10.1371/journal.pone.0005808)

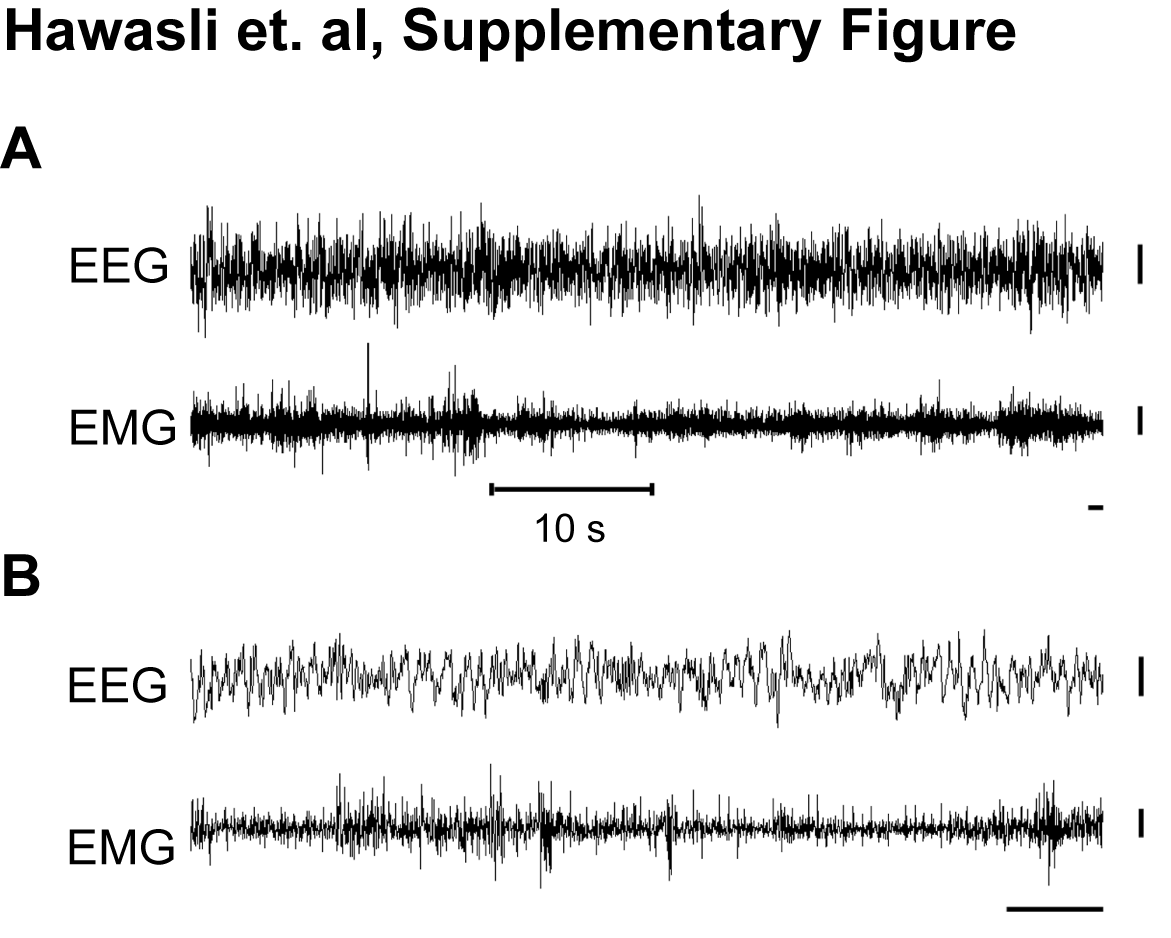

Supplement: Figure S1 — Representative EEG/EMG recordings from a wild-type control mouse. A, A period of normal wakefulness. B, Expanded view of 10 sec of the recording period shown in A. Calibration 1 sec and 50 µV; the recording period shown in panel B is annotated by the 10 sec bar in A. (0.19 MB TIF) [file pone.0005808.s001.tif]
